# Quality Factor Effect on the Wireless Range of Microstrip Patch Antenna Strain Sensors

**DOI:** 10.3390/s140100595

**Published:** 2014-01-02

**Authors:** Ali Daliri, Amir Galehdar, Wayne S. T. Rowe, Sabu John, Chun H. Wang, Kamran Ghorbani

**Affiliations:** 1 School of Electrical and Computer Engineering, RMIT University, GPO Box 2476, Melbourne, Victoria 3001, Australia; E-Mails: amir.galehdar@dsto.defence.gov.au (A.G.); wayne.rowe@rmit.edu.au (W.S.T.R.); kamran.ghorbani@rmit.edu.au (K.G.); 2 School of Aerospace, Mechanical and Manufacturing Engineering, RMIT University, PO Box 71, Bundoora, Victoria 3083, Australia; E-Mails: sabu.john@rmit.edu.au (S.J.); chun.wang@rmit.edu.au (C.H.W.)

**Keywords:** wireless strain sensor, microstrip patch antenna, quality factor, structural health monitoring, passive sensor

## Abstract

Recently introduced passive wireless strain sensors based on microstrip patch antennas have shown great potential for reliable health and usage monitoring in aerospace and civil industries. However, the wireless interrogation range of these sensors is limited to few centimeters, which restricts their practical application. This paper presents an investigation on the effect of circular microstrip patch antenna (CMPA) design on the quality factor and the maximum practical wireless reading range of the sensor. The results reveal that by using appropriate substrate materials the interrogation distance of the CMPA sensor can be increased four-fold, from the previously reported 5 to 20 cm, thus improving considerably the viability of this type of wireless sensors for strain measurement and damage detection.

## 1. Introduction

In the last few decades, technological advances and state-of-the-art engineered materials have enabled researchers and engineers to develop several novel methods for Structural Health Monitoring (SHM). These methods are based on a variety of physical phenomena such as eddy current [1], thermal/infrared [2,3] and electromagnetics [4,5]. In addition, sensor based health and usage monitoring systems utilize several types of sensors to monitor strain or crack growth in the structure. These sensors include resistive strain gauges, piezoelectric transducers, fiber optic sensors, and many more. However, the required wiring to connect and power these sensors is a major issue for the broad adoption of SHM. Wireless strain sensors, powered by an external power source/integrated battery unit, have been extensively studied in the literature. The complexity, large size, added weight, and the limited lifetime of batteries (power source) restrict the integration of such sensors in SHM systems.

More recently, the concept of passive wireless strain sensors based on various types of electromagnetic resonators has been introduced to reduce the complexity of traditional wireless sensors by eliminating the need for integrated communication components and power sources [6–16]. Details on the different methods used for developing passive wireless sensors for SHM can be found in two recent review articles [15,16]. Such electromagnetic structures are promising candidates for wireless SHM as their resonant frequency, which is sensitive to their physical dimensions, can be exploited for measurement. These structures include microstrip patch antennas [6–10], Radio Frequency Identification (RFID) tags [11,12], and metamaterial inspired resonators [13,14].

Circular microstrip patch antennas (CMPAs) are one of the promising devices to measure strain in different aerospace materials (both conductive and non-conductive); a linear relationship exists between the strain and the shift in their resonant frequency [8–10]. Recently, a technique to monitor the resonant frequency of CMPAs wirelessly using a linearly polarized horn antenna was demonstrated numerically and experimentally [17]. It was shown that using this technique strain could be monitored in any desired direction by rotating the horn antenna because the CMPA was excited in the direction aligned to the polarization plane of the horn antenna [17]. An important parameter in the wireless measurement of strain using CMPAs is the interrogation distance between the reader antenna and the CMPA sensor. It was reported that by increasing the interrogation distance, the reliability of measuring the shift in the resonant frequency of the CMPA decreases; the maximum practical interrogation distance was found to be 5 cm [17].

In the case of metamaterial resonators [14] and inductive coupling of LC circuits [18] the interrogation distance is limited to a few centimetres. For RFID-based sensors the maximum interrogation distance that has been reported was 1.270 m where curve fitting was required to determine the resonant frequency [12]. For these sensors an additional Integrated Circuit (IC) chip is required which increases the size and complexity of the sensor unit. The maximum interrogation distance for microstrip patch antennas reported in the literature is 1 m where an additional light activated RF switch is required to separate the sensor response from the structural response [6]. In a recent study using dipole antennas as the sensing element the maximum interrogation distance reported is 15 cm [19]. However, the dipole element has been attached to a dielectric where the reflections from the structure are not significant. Until now, no detailed study of the effect of antenna design parameters on the interrogation distance of passive antenna strain sensors, without using additional circuit elements, has been reported.

The wireless reading range of CMPAs needs to be increased to enable the practical implementation of this technique for SHM. In this paper, we present a study of the effect of CMPA's quality factor (which is a representation of the losses in the CMPA) on the interrogation distance. First, the quality factor of microstrip patch antennas and the effect of various antenna parameters on the quality factor are studied. Based on this study, a CMPA with high quality factor is designed and the effect of antenna quality factor on the reading range is discussed. Finally, the CMPA with high quality factor is fabricated and its improved performance in wireless strain measurement is validated experimentally. The results show that by using high quality factor antennas/resonators the wireless reading range can be increased significantly.

## 2. Theoretical Considerations and Computational Analyses

The fractional bandwidth of an antenna is inversely proportional to the quality factor of the antenna and it can be expressed as [20]:

(1) $$\text{Fractional Bandwidth}=\frac{\Delta f}{f}=\frac{1}{Q_{t}}$$

A more precise relationship can be defined by accounting for the impedance matching of the antenna [20]. However, for the wireless excitation of a microstrip patch antenna this impedance matching dependence is not required because the patch antenna is not excited using a transmission line (such as a coaxial cable). The quality factor of an antenna can be defined as a representation of the antenna losses [20]. According to [21] the total quality factor for a circular microstrip patch antenna can be calculated from the following equation:

(2) $$\frac{1}{Q_{t}}=\frac{1}{Q_{\text{rad}}}+\frac{1}{Q_{c}}+\frac{1}{Q_{d}}+\frac{1}{Q_{sw}}$$

**Figure 1.**
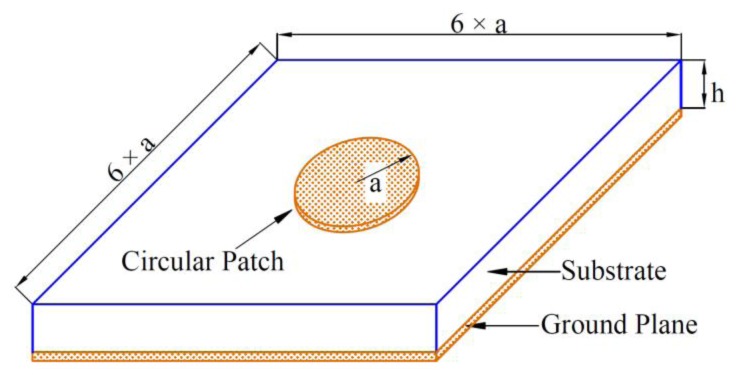
Geometry of the CMPA sensor.

Figure 1 shows the geometry of the CMPA sensor. For very thin substrates (*h*<<*λ_0_*), the loss due to surface waves, 1/*Q_sw_*, is very small and can be neglected in calculation of the total quality factor [21]. This indicates that by reducing the thickness of the patch antenna the quality factor due to the surface waves can be improved and as a result of that the total quality factor of the antenna can be improved. The other quality factors can be calculated using following equations [21]:

(3) $$Q_{c}=h\sqrt{\pi f\mu_{0}\sigma }$$

(4) $$Q_{d}=\frac{1}{\tan\delta }$$

and for the dominant mode of operation [22]:

(5) $$Q_{\text{rad}}=\frac{30[{\left(ka\right)}^{2}-1]}{hf\mu_{0}{\left(k_{0}a\right)}^{2}I_{1}}$$

where:

(6) $$I_{1}=\int_{0}^{\pi /2}[{J_{1}}^{\prime 2}(k_{0}a\sin\theta )+\cos^{2}\theta {J_{1}}^{2}(k_{0}a\sin\theta )/{\left(k_{0}a\sin\theta \right)}^{2}]\sin\theta d\theta$$

The quality factor due to dielectric losses can be improved by using low loss materials for the antenna substrate. From Equation (3), the conductive loss can be reduced by increasing the conductivity of the patch and the ground plane. In practice, the *Q_c_* will be lower than Equation (3) because of the surface roughness of the patch and the ground plane. According to [20], for very thin substrates the dominant factor is the radiation quality factor. This part of the total quality factor is proportional to the substrate dielectric constant and the inverse of the substrate thickness.

### 2.1. Effect of Substrate Material on Quality Factor

In order to improve the quality factor of the CMPA, a numerical investigation into the effect of each parameter of the total quality factor was carried out. To this end, two commercially available substrates were selected, namely a FR4 (ε*_r_* = 4.5, tanδ = 0.025) and Rogers™ RT/duroid 6010.2LM™ (ε*_r_* = 10.2, tanδ = 0.0023 [23]; Rogers Corporation, Brooklyn, CT, USA). The FR4 substrate was selected because it was used in previous studies on CMPA sensors [8–10,17] and the Rogers™ substrate (which is called high Q material in the rest of the paper) was used because it has much lower tangent loss and much higher permittivity compared to FR4. For the numerical study the thickness of each substrate was varied within the range of commercially available laminate thicknesses, *i.e.*, 0.127, 0.254, 0.635, 1.27, 1.90, 2.50 mm for high Q material and 0.8, 1.0, 1.2, 1.5, 2.0, 2.4 mm for FR4. The antennas were designed to resonate at 1.5 GHz (similar to previous studies on CMPAs [8–10,17]).

**Figure 2.**
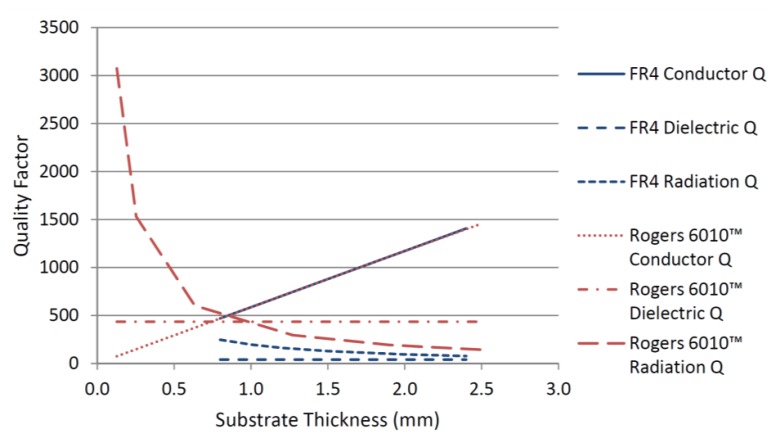
Conductor, dielectric and radiation quality factors for two substrate materials studied with different thicknesses.

The conductor, dielectric and radiation quality factors for the two substrates were calculated using Equations (3)–(6) and the results for different substrate thicknesses are shown in Figure 2. The results show that the conductor quality factor is independent of the substrate permittivity and varies linearly with the substrate thickness. From Equation (4), the dielectric quality factor depends only on the loss tangent of the substrate. The results in Figure 2 shows for high Q material the dielectric quality factor is much higher than the FR4 substrate. The radiation quality factor depends on both the substrate thickness and the substrate material's permittivity. The results in Figure 2 show that radiation quality factor is higher for the substrate with the higher dielectric constant regardless of the substrate thickness. By reducing the substrate thickness of the high Q material the radiation quality factor increases dramatically.

**Figure 3.**
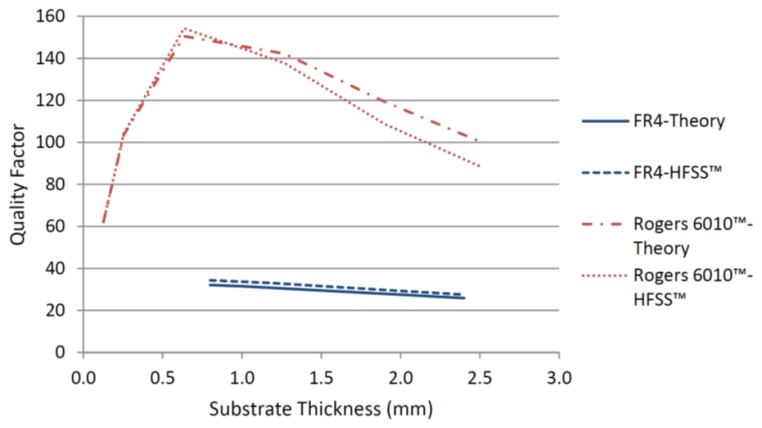
Total quality factor for the two substrates studied at different thicknesses.

Figure 2 indicates that the substrate with higher permittivity and lower loss tangent will always have a higher total quality factor regardless of the substrate thickness. This can also be seen from Figure 3 where the total quality factors for the two substrates, according to Equation (2), are compared with the numerical results from HFSS™ (Eigen mode solver) (ANSYS, Canonsburg, PA, USA) [24]. Substrate thickness has opposite effects on the radiation and conductor quality factors. Figure 3 shows that for commercially available thicknesses of high Q material, the optimum thickness to achieve the highest total quality factor is 0.635 mm. Therefore, this thickness was chosen for further simulations and fabrication of the CMPA sensor to be employed in experimental measurements.

### 2.2. Effect of Quality Factor on Interrogation Distance

**Figure 4.**
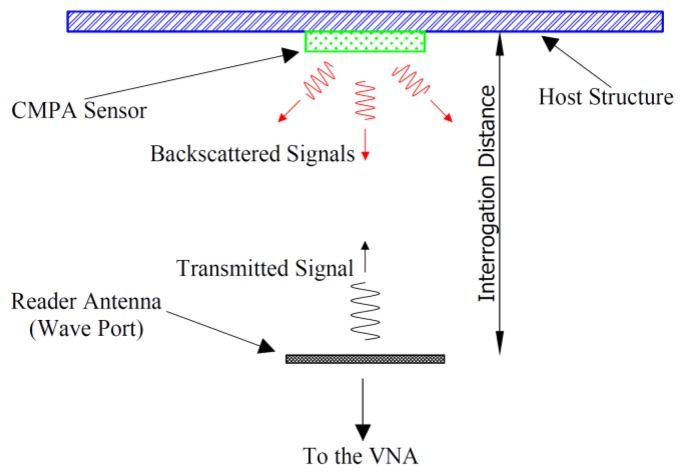
Schematic of the wireless setup for simulation and measurement.

**Figure 5.**
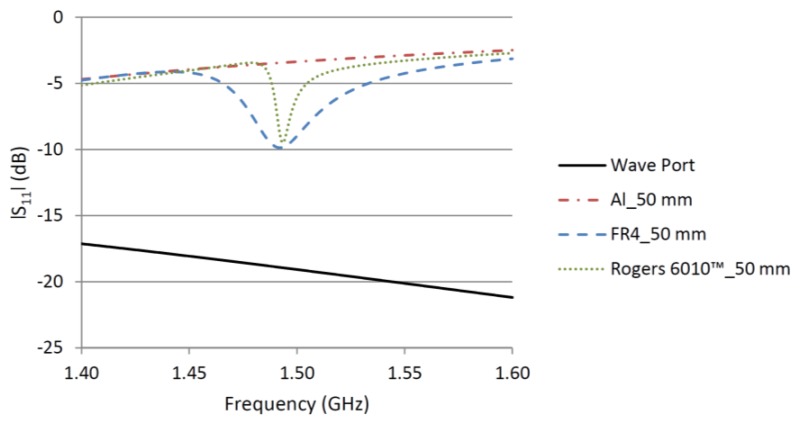
Effect of quality factor on the |S_11_| of two CMPAs.

To investigate the effect of the quality factor on the interrogation distance of a CMPA strain sensor, two CMPAs were analyzed in HFSS™ with a setup similar to the simulation schematic in [17]. To reduce the computational time, the horn antenna used in [17] was replaced with a rectangular wave port with the same aperture where only mode 1 of the wave port was excited. Figure 4 shows the diagram of the setup used in the simulations and measurements. Figure 5 presents the results of |S_11_| of the wave port for the interrogation distance of 5 cm when CMPA was attached to an aluminum plate and placed in front of the wave port. In addition, the |S_11_| of the wave port without an object and with an aluminum plate in front of it are shown.

As discussed in [17], the reflection from the aluminum plate increases the |S_11_| at the wave port because some of the incident energy is reflected back to the port. The magnitude of the |S_11_| depends on the distance between the aluminum plate and the wave port resulting from a standing wave. Regardless of the |S_11_| magnitude for the aluminum plate, by attaching a CMPA to the aluminum plate the resonant frequency of the CMPA can be clearly distinguished. By increasing the interrogation distance the difference between the |S_11_| magnitude from the bare aluminum plate and the |S_11_| at the resonant frequency of the CMPA become less, which makes it difficult to detect the resonant frequency of the CMPA. Figure 5 shows that for the same interrogation distance and same resonant frequency, the dip of |S_11_| is much sharper (meaning the CMPA bandwidth is smaller) for the high Q substrate compared to the FR4 design. This is the effect of the higher quality factor of the CMPA designed using the high Q material. The sharper response of the high Q CMPA results in easier and more accurate tracking of the shift of the CMPA resonant frequency due to strain, because the sharper response has one clear minimum.

**Figure 6.**
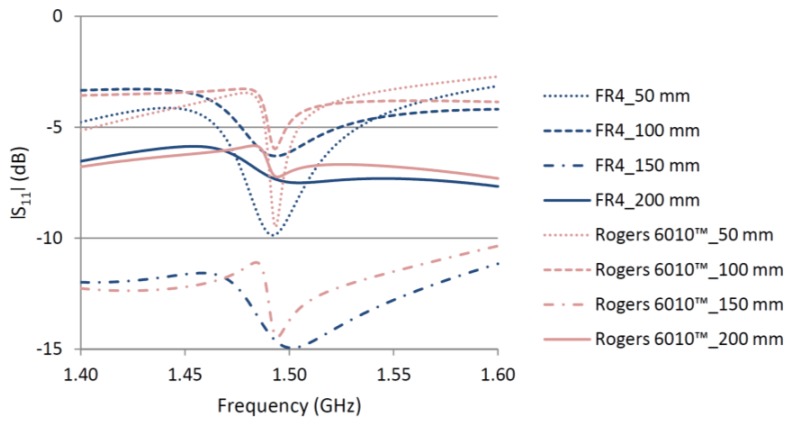
Effect of interrogation distance on the |S_11_| of two CMPAs.

Figure 6 shows the effect of interrogation distance on the |S_11_| of both CMPAs attached to the aluminum plate. By changing the interrogation distance the standing wave between the aluminum plate and the wave port changes and accordingly affects the magnitude of the |S_11_|. However, regardless of the level of |S_11_| for the aluminum plate, the resonant frequency of the CMPA generates a dip in the |S_11_| curve. The relative magnitude of this dip decreases with the interrogation distance for both CMPAs. Beyond a certain interrogation distance this relative dip vanishes, making it very difficult to detect the resonant frequency of the CMPA. Figure 6 shows that for the CMPA designed with high Q material because of a sharper dip in the |S_11_| curve the resonant frequency of the CMPA can be distinguished from the structure response up to 20 cm whilst for the CMPA designed with FR4 it is more difficult to distinguish the resonant frequency even at 10 cm.

## 3. Experimental Measurements

To validate the feasibility of increasing wireless reading range using high quality factor CMPAs, the proposed high Q CMPA was fabricated and its strain response was measured. The experimental setup was similar to that employed in [17]. A linearly polarized horn antenna (DRH118™ horn antenna from Sunol Sciences^®^ [25]) (Sunol Sciences Corporation, Dublin, CA, USA) was used as the reader antenna. In order to apply strain to the host structure, a tensile force of 20 kN was applied to the assembly in 20 load increments (1 kN steps) using a 100 kN INSTRON™ machine (Instron, Norwood, MA, USA). At the maximum applied load, the strain in the center of the host structure is 500 με. Nine conventional resistive strain gauges were attached to the back of the host structure to verify that a uniform strain is applied in the region where the CMPA is attached. The |S_11_| of the horn antenna and the strain were measured at each loading step. The resonant frequency of the CMPA has been extracted by reading the minima in the |S_11_| curve as well as the smith chart (real and imaginary parts). The network analyzer was set to measure 1601 points in the measurement frequency range, resulting in a resolution of 25 με per 25 kHz increment. Due to slight differences in the resonant frequencies, the measurement frequency range for the FR4 and high Q CMPAs were 1.535 GHz to 1.575 GHz, and 1.470 GHz to 1.510 GHz, respectively. Reducing the frequency increments below 25 kHz was found to produce no improvement in the sensitivity of the sensor.

**Figure 7.**
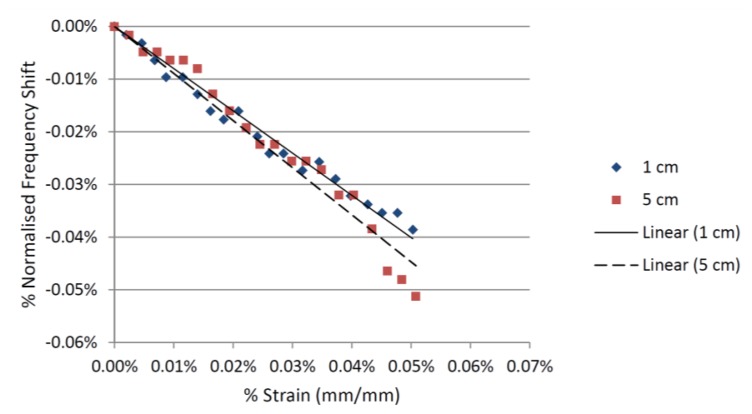
Strain-frequency shift relationship for the FR4 CMPA sensor.

For both CMPAs, the tests were conducted for interrogation distances of 1, 5, 10, 15 and 20 cm. The wireless measurement of strain for the CMPA made from FR4 was only possible at interrogation distances of up to 5 cm. By increasing the interrogation distance the |S_11_| curve de-stabilizes and the minima moves back and forth. These variations in the |S_11_| were greater than the shift in the resonant frequency (which is caused by the applied strain). This resulted in a decrease in the signal to noise ratio of the wireless strain measurement. The relationships between the strain and the shift in the resonant frequency of the CMPA with FR4 substrate at two different interrogation distances are shown in Figure 7. A linear relationship between the applied strain and the shift in the resonant frequency is observed for 1 and 5 cm interrogation distances. At interrogation distances beyond 5 cm wireless strain measurement was no longer possible for the CMPA made from FR4, because of the increased variations in the |S_11_|.

**Figure 8.**
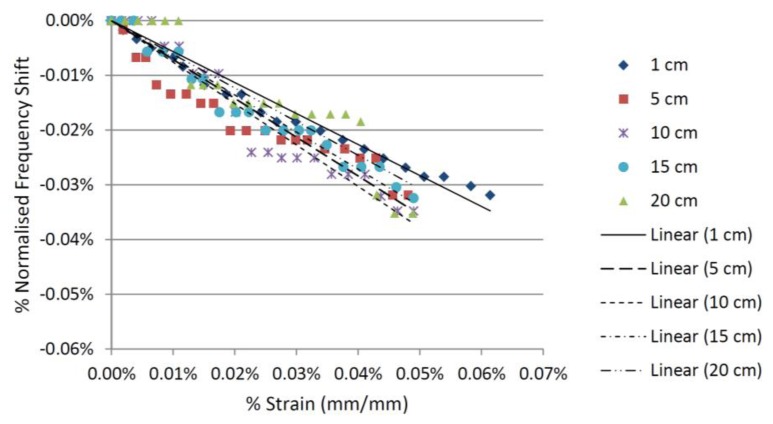
Strain-frequency shift relationship for the Rogers 6010™ CMPA sensor.

For the CMPA made from high Q material the wireless strain measurement remained reliable at interrogation distances of up to 20 cm, albeit with a slight decrease in resolution at longer distances. Figure 8 shows the relationship between the strain and the shift in the resonant frequency of the CMPA with the high Q material at interrogation distances of 1, 5, 10, 15 and 20 cm. The results for the 20 cm interrogation distance show the increased wireless reading range using this substrate, confirming the findings from the computational simulation presented in the previous section. Therefore, utilizing a substrate with higher permittivity and lower loss tangent with the right thickness improves the wireless range of the CMPA because of increased quality factor. It is envisaged that increasing gain and efficiency of the antenna sensor and the reader antenna will also improve the interrogation distance.

## 4. Conclusions

The effect of quality factor on the interrogation distance of CMPA strain sensors was investigated using numerical calculations and experimental measurements. The results presented in this paper show that the quality factor of the CMPA has a significant effect on the wireless reading range. Strain measurement at interrogation distances up to 20 cm was possible using the high Q material where previous studies using FR4 CMPA were only able to measure strain at 5 cm. Therefore the interrogation distance can be increased by a factor of 4 when a substrate with lower loss and higher permittivity is employed. The analysis presented in this paper was based on commercially available substrates. Further increase in the interrogation distance is possible by using specially designed materials that exhibit significantly low losses and very high dielectric constants.

## Nomenclature

*Q_t_*: the total quality factor of the antenna
*Q_rad_*: the quality factor of the antenna due to radiation losses
*Q_c_*: the quality factor of the antenna due to conductive losses
*Q_d_*: the quality factor of the antenna due to dielectric losses
*Q_sw_*: the quality factor of the antenna due to surface waves
*h*: the substrate thickness
*f*: the resonant frequency of the antenna
Δ*f*: the frequency difference
μ_0_: the permeability of free space
ε*_r_*: the substrate relative permittivity
λ_0_: the wavelength in free space
σ: the conductivity of the metal used for the patch and ground plane
tanδ: the loss tangent of the substrate
*k*: the wave number in the substrate
*k*_0_: the wave number in free space
*a*: the radius of the CMPA
*J*_1_: the Bessel function of the first kind of order one
${J_{1}}^{\prime }$: the derivative of *J*_1_
